# Foodborne botulism presenting as small bowel obstruction: a case report

**DOI:** 10.1186/s12879-020-05759-0

**Published:** 2021-01-12

**Authors:** Alberto Friziero, Cosimo Sperti, Gianfranco Da Dalt, Nicola Baldan, Gianpietro Zanchettin, Pasquale Auricchio, Laura Gavagna, Andrea Grego, Giulia Capelli, Stefano Merigliano

**Affiliations:** Department of Surgery, Oncology and Gastroenterology, 3rd Surgical Clinic, University of Padua, via Giustiniani 2, 35128 Padova, Italy

**Keywords:** Case report, Botulism, Foodborne botulism, Clostridium botulinum, Small bowel obstruction

## Abstract

**Background:**

Small bowel obstruction is one of the leading reasons for accessing to the Emergency Department. Food poisoning from *Clostridium botulinum* has emerged as a very rare potential cause of small bowel obstruction. The relevance of this case report regards the subtle onset of pathognomonic neurological symptoms, which can delay diagnosis and subsequent life-saving treatment.

**Case presentation:**

A 24-year-old man came to our Emergency Department complaining of abdominal pain, fever and sporadic self-limiting episodes of diplopia, starting 4 days earlier. Clinical presentation and radiological imaging suggested a case of small bowel obstruction. Non-operative management was adopted, which was followed by worsening of neurological signs. On specifically questioning the patient, we discovered that his parents had experienced similar, but milder symptoms. The patient also recalled eating home-made preserves some days earlier. A clinical diagnosis of foodborne botulism was established and antitoxin was promptly administered with rapid clinical resolution.

**Conclusions:**

Though very rare, botulism can mimic small bowel obstruction, and could be associated with a rapid clinical deterioration if misdiagnosed. An accurate family history, frequent clinical reassessments and involvement of different specialists can guide to identify this unexpected diagnosis.

## Background

Small bowel obstruction (SBO) is one of the leading reasons for accessing to the Emergency Department with abdominal complaints, accounting for 12–16% of hospital admissions for acute abdominal pain in the US [1]. The management of this condition depends strongly on its pathogenesis and clinical presentation. According to the Bologna Guidelines for the diagnosis and management of adhesive small bowel obstruction (ASBO), a non-surgical management should be adopted when no clear cause of obstruction is identified, and in the absence of clinical and radiological signs of acute conditions requiring emergency surgery (i.e. strangulation, bowel ischemia or peritonitis) [2].

.Food poisoning from *Clostridium botulinum* has emerged as a very rare potential cause of SBO. In 2017, there were 182 laboratory-confirmed cases of botulism reported to the Atlanta Center for Disease Control, 19 (10%) of which were foodborne [3]. In Italy, there were 421 confirmed cases of foodborne botulism between 1986 and 2015 [4]. Its clinical presentation usually includes both gastrointestinal and neurological symptoms, manifesting 12–72 h after ingestion of the contaminated food [5]. Gastrointestinal symptoms mainly include nausea, vomiting and non-specific, diffuse abdominal pain. Early-onset neurological symptoms include ptosis, disturbed vision, dilated and fixed pupils, dysphagia, dry mouth and dysphonia, which can progress to a descending symmetrical flaccid paralysis.

Herein we present a clinical case of foodborne botulism presenting with fever and abdominal symptoms of SBO, associated with mild and discontinuous neurological symptoms (lethargy and intermittent diplopia).

We reviewed the English literature, using PubMed (Medline), EMBASE and Scopus, to retrieve previously-reported cases of botulism initially presenting with clinical and radiological signs of SBO. As keywords we used: “small bowel obstruction”, “botulism”, and “botulinum”. The “related articles” function was used to widen the search, and all abstracts, studies, and citations retrieved were reviewed. We found only one published case, featuring a previously-healthy 45-year-old man, who underwent emergency laparotomy for clinically and radiologically suspected SBO [6].

.This case report is relevant because it shows an unusual clinical presentation of botulism, with a subtle onset of pathognomonic neurological symptoms, which - especially in an area in which very few cases have been reported - can delay diagnosis and subsequent life-saving treatment with antitoxins.

## Case presentation

A 24-year-old man came to our Emergency Department complaining of abdominal pain and fever starting 4 days earlier.

The previously-healthy patient arrived at the Emergency Department with subacute-onset abdominal distension and mildly blurred vision, with intermittent, self-limiting episodes of diplopia. The patient reported no nausea or vomiting, and a regular bowel function.

At initial assessment, the patient was feverish (body temperature 38 °C), hemodynamically stable, mildly lethargic, but with no signs of neurological impairment, and he scored 15 on the Glasgow Coma Scale.

Physical examination of the abdomen confirmed mild distension, with rebound tenderness in the right iliac fossa, and a slightly positive Blumberg sign.

Blood tests showed no major alterations other than a high C- reactive protein (CRP) level of 86.3 mg/L (normal range 0.0–3.0 mg/L).

An oropharyngeal swab for the detection of SARS-CoV-2 (necessary to allow for further diagnostic and therapeutic procedures) was negative.

**Fig. 1:**
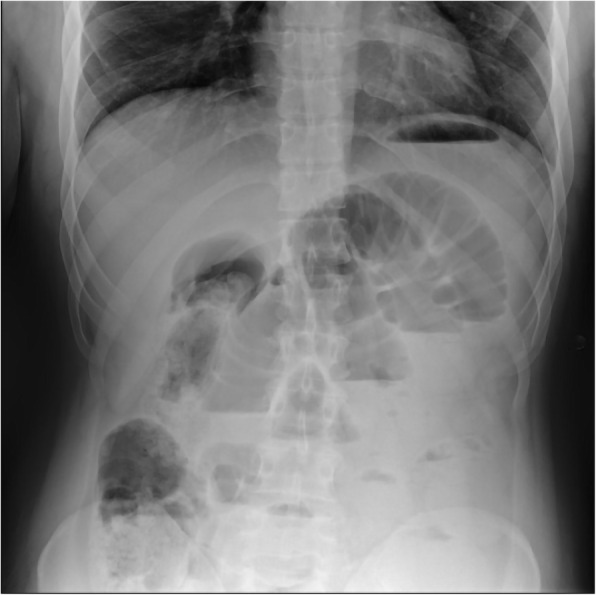
Abdominal X-rays showing small bowel distension with air-fluid levels

Abdominal X-rays showed small bowel distension and the presence of air-fluid levels. (Fig. 1).

**Fig. 2:**
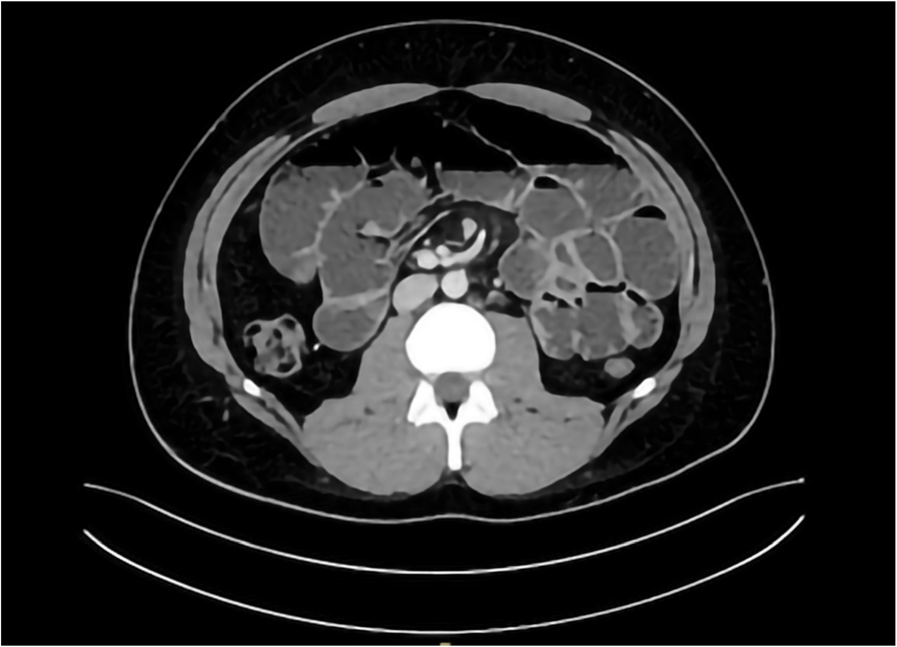
CT scan with intravenous contrast showing small bowel distension with air-fluid levels, and a reduced distal ileum diameter in the right iliac fossa

A subsequent abdominal computed tomography (CT) scan with intravenous contrast confirmed the small bowel distension with air-fluid levels, and showed a reduction in distal ileum diameter in the right iliac fossa with an empty marbled colon. No obvious causes of mechanical SBO came to light. (Fig. 2).

A brain CT scan was also performed to exclude any organic causes of diplopia, and revealed no relevant alterations.

Since no clear cause of mechanic obstruction was identified, and the patient had no signs or symptoms of peritonitis, we opted for a conservative management in accordance with current guidelines [2].

**Fig. 3:**
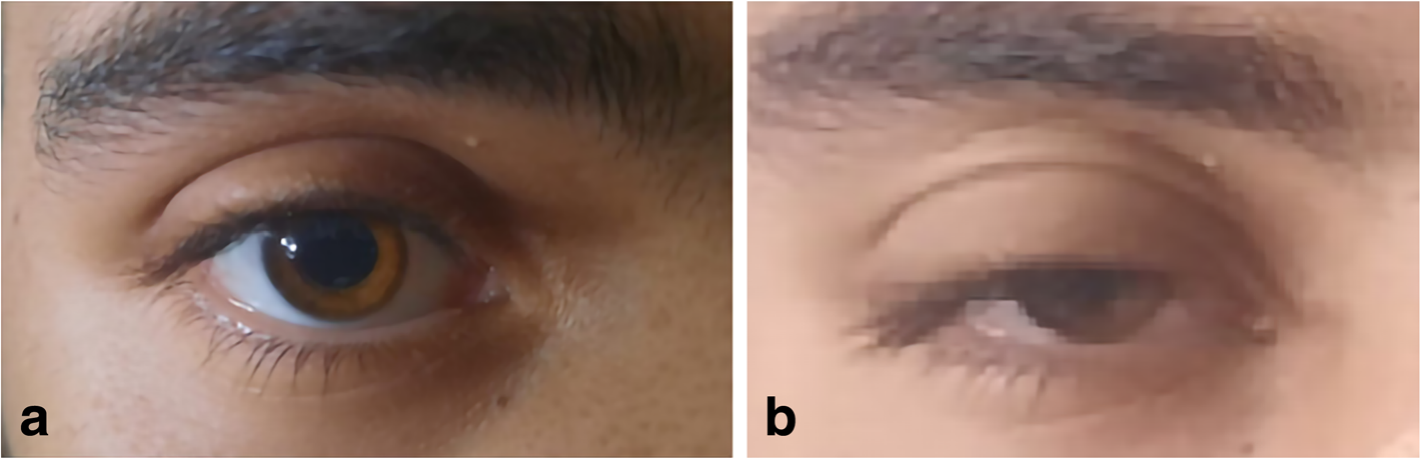
Neurological signs. **a** Mydriasis. **b** Ptosis

A nasogastric tube was inserted and the patient received nil per os with intravenous supportive therapy. Twenty-four hours after admission, his abdominal pain had improved, and clinical examination showed a pain-free, less distended abdomen. Meanwhile, his neurological signs had worsened, with xerostomia, ptosis, mydriasis and diplopia. (Fig. 3a and b) We therefore had the patient assessed by our consultant neurologist. Both heavy metal and food poisoning were considered in the differential diagnosis. On specifically questioning the patient, we discovered that his parents had experienced similar, but milder symptoms, such as dizziness, xerostomia, nausea and non-specific, diffuse abdominal pain; anyhow, no signs of SBO were present in the patient’s parents. The patient also recalled eating home-made preserves 6 days earlier. After consulting our Poison Control Center, i.e. the Centro Antiveleni (CAV) e Centro Nazionale di Informazione Tossicologica (C.N.I.T.) of Pavia, a clinical diagnosis of foodborne botulism poisoning due to home-made preserves was formulated. The clinical suspicion was confirmed by multiplex real-time polymerase chain reaction (CNRB31.0112019 Rev.1) assay [7], performed at the Istituto Superiore di Sanità (ISS), Dipartimento di Sicurezza Alimentare, Nutrizione e Sanità Pubblica Veterinaria, to which all suspected botulism cases are referred for laboratory confirmation. Five consecutive rectal swabs were positive for *Clostridium botulinum type B*.

We consequently started treatment with Botulism Antitoxin Heptavalent (BAT®), obtaining a near-total resolution of the patient’s symptoms within about 24 h.

The patient’s parents also tested positive for *Clostridium botulinum type B*, with the same diagnostic modalities mentioned above, and were treated accordingly, obtaining immediate clinical benefit.

## Discussion and conclusions

SBO is one of the leading causes of hospital admissions for surgery, and one of the most common emergencies requiring surgical treatment [8]. Although postoperative or post-infectious adhesions are responsible for most cases of SBO, no mechanical cause of the obstruction can be identified in about 11% of cases [9]. This possibility should not be underestimated especially in emergency surgery units, where large numbers of cases of SBO are treated every year. In this setting, even unusual or rare diseases should be included in the differential diagnosis of SBO with no apparent mechanical cause.

To date, six forms of botulism are recognized and classified according to the modality of exposure to the toxin [4].

.The last relevant outbreak of food borne botulism in Italy dates back to 1996 [10]. Nevertheless, such a possible diagnosis should not be neglected when faced with patients presenting acute gastrointestinal complaints associated with even mild neurological symptoms.

Only one case had previously been reported in the English literature: Authors described performing an emergency exploratory laparotomy in a patient presenting with SBO, who was subsequently diagnosed with botulism. No data were available concerning the patient’s postoperative recovery, or the effects of general anesthesia and curarization in particular [6].

.As concerns other forms of botulism, it is acknowledged that intestinal botulism (due to intestinal colonization by neurotoxigenic clostridia) can mimic a surgical emergency [11]. Three cases have been reported of intestinal botulism in adult patients presenting with acute abdominal symptoms [12]. Two of them underwent surgery for clinically-suspected acute appendicitis, and both experienced worsening neurological symptoms after surgery, subsequently requiring intensive care with mechanical ventilation. The third patient was admitted for neurological symptoms (i.e. diplopia and dysphagia), nausea and vomiting, and was treated conservatively, with a more favorable outcome. It is therefore reasonable to believe that the unnecessary surgical treatment of misdiagnosed botulism could be associated with a higher rate of postoperative complications, including the need for mechanical ventilation.

In our case, frequent clinical reassessments provided the key to identifying new, more relevant symptoms pointing to an unexpected diagnosis. The involvement of consultants from different specialties also contributed to improve the patient’s final outcome. It is worth noting that the patient’s family history provided an important clue to the diagnosis; the significance of this aspect when collecting a patient’s history should never be underestimated.

In conclusion, it is important to acknowledge that the extensive use of standardized protocols for managing the most common surgical emergencies can sometimes be misleading when we are dealing with an uncommon diagnosis. Though very rare, botulism has the potential to cause SBO, and could be associated with a rapid clinical deterioration if it goes misdiagnosed. Conducting frequent clinical reassessments, involving specialists from different fields, and paying careful attention to the possibility of less common diagnoses could be the key to a patient’s positive outcome.

## Data Availability

Not applicable.

## Abbreviations

SBO: Small bowel obstruction
ASBO: Adhesive small bowel obstruction
CRP: C-reactive protein
CT: Computed tomography
SARS-CoV-2: Severe acute respiratory syndrome coronavirus 2
